# Photonics and fracture toughness of heterogeneous composite materials

**DOI:** 10.1038/s41598-017-04782-7

**Published:** 2017-07-03

**Authors:** S. Joseph Antony, George Okeke, D. Deniz Tokgoz, N. Gozde Ozerkan

**Affiliations:** 1 School of Chemical and Process Engineering, University of Leeds, Leeds, LS2 9JT UK; 2 Center for Advanced Materials, Qatar University, P.O. Box 2713, Doha, Qatar

## Abstract

Fracture toughness measures the resistance of a material to fracture. This fundamental property is used in diverse engineering designs including mechanical, civil, materials, electronics and chemical engineering applications. In spite of the advancements made in the past 40 years, the evaluation of this remains challenging for extremely heterogeneous materials such as composite concretes. By taking advantage of the optical properties of a thin birefringent coating on the surface of opaque, notched composite concrete beams, here we sense the evolution of the maximum shear stress distribution on the beams under loading. The location of the maximum deviator stress is tracked ahead of the crack tip on the experimental concrete samples under the ultimate load, and hence the effective crack length is characterised. Using this, the fracture toughness of a number of heterogeneous composite beams is evaluated and the results compare favourably well with other conventional methods using combined experimental and numerical/analytical approaches. Finally a new model, correlating the optically measured shear stress concentration factor and flexural strength with the fracture toughness of concretes is proposed. The current photonics-based study could be vital in evaluating the fracture toughness of even opaque and complex heterogeneous materials more effectively in future.

## Introduction

Concrete is one of the most complex group of heterogeneous engineering materials used abundantly in the world. Normally it consists of sand, cement and gravel at different sizes, and consumes water in the fabrication of concrete structures^1^. To improve the mechanical properties of concretes and reduce the usage of natural aggregates, other host materials such as fibre reinforcements^2^, cementitious materials^3^ and grains derived from the municipal wastes as partial substitutes for aggregates^4, 5^ are being utilised in the construction sector. This makes standardising the mechanical analysis of such heterogeneous materials even more challenging. Using the novel principles of photonics, we focus here on evaluating the fracture toughness^6^ of heterogeneous concrete mixes in which recycled municipal wastes are used as partial replacement of aggregates^5^.

In spite of the continuous improvements made on the standards and methodology of evaluating the fracture toughness of different materials over the past 40 years, accurate estimates of fracture toughness for complex mixes of concretes still remain as a stiff task^7, 8^.

**Figure 1:**
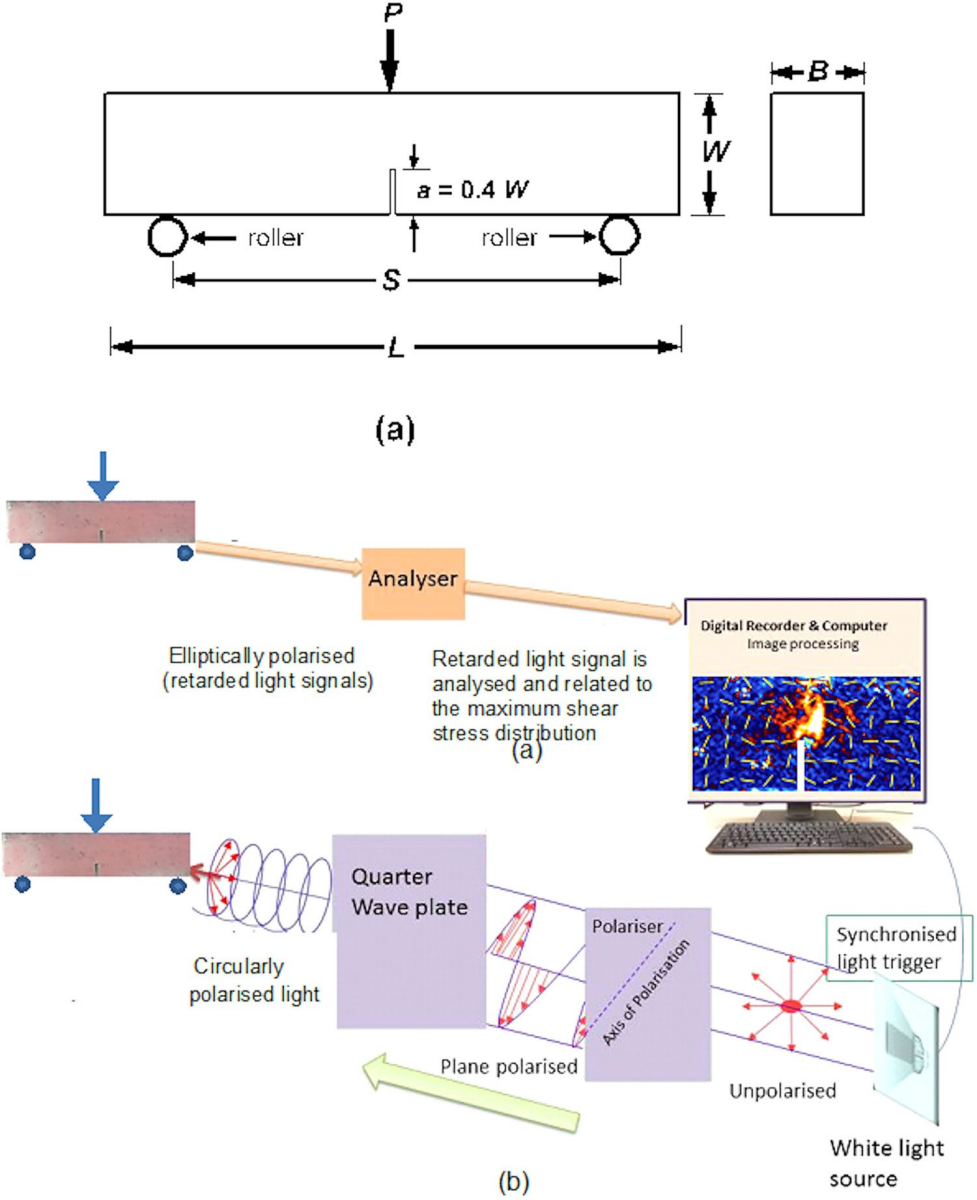
Schematic diagrams of (**a**) three-point bending test and (**b**) photo stress analysis tomography experimental setup.

At the present time, fracture toughness of concretes is evaluated using a combination of analytical^9^, numerical^10^ and experimental methods^6^. Fracture mechanics-based concepts, such as LEFM (Linear Elastic Fracture Mechanics), have been developed to determine the fracture strength of concrete^11^. In this, the material fracture occurs when the crack stress intensity factor reaches its critical value, namely the critical stress intensity factor *k*_*c*_, corresponding to the fracture energy *G*_*c*_^7, 8^. Fracture mechanics could describe both the linear and nonlinear fracture behaviour of concretes^12, 13^. For the experimental measurement of fracture toughness such as using ASTM 1820^14^, notched concrete beam samples (with a pre-fabricated notch) are used to evaluate the conservative value of the fracture toughness, which serves as a limiting value in the engineering designs^13, 14^. The estimates of this can also account for the size effects of the concrete geometry (beam)^15^. Basically, the commonly used methods such as the two-parameter model^16^, effective crack(notch) model^17^ and the effective crack notch model with consideration to the mode 1 shear stress field (ECMS, methods section) depends on the features of the crack-processing zone ahead of the notch tip^17^. The effective crack depth accounts for the location of this crack processing zone ahead of the notch tip (in addition to the initial depth of the notch (Fig. 1)). In general, this is not easy to determine for the complex heterogeneous composite mixes. In the above said models^13, 17^, the effective crack depth is determined in conjunction with finite element method^18^ or regression analysis^17, 18^ involving a number of regression coefficients obtained from several experimental trials for a given concrete mix (methods section). Conceptually, the crack processing zone is where deviator stress^19^ is dominant ahead of the notch tip, and the experimental determination of this is cumbersome. For transparent birefringent continuum materials, photoelastic analysis has been used to determine the stress intensity factor *K*_*IC*_ ahead of the crack tip using the characteristics of optical fringes^20^, which is then correlated to the fracture toughness^20, 21^. However, the evaluation of this for opaque and heterogeneous concretes still remains as very challenging. This is primarily due to the difficulties in sensing the birefringent properties on such opaque materials, as well as the nature of the distribution of the fringes could be irregular in shape in heterogeneous composites. The current work aims to address these challenges elegantly.

## Experiments

In the current study, seven types of notched concrete samples were used to evaluate their fracture toughness. In brief, seven mixes were prepared with steel fibre, fly ash, silica fume as well as virgin and recycled low and high density polyethylene (LDPE and HDPE respectively) in different proportions. The fly ash used was obtained from the Qatar’s Domestic Solid Waste Management Centre’s (DSWMC) flue gas treatment system for recycling the domestic wastes. Similarly, virgin LDPE and HDPE were obtained from the Qatar Chemical Company (QCHEM) and Qatar Petrochemical Company (QAPCO) respectively. The virgin LDPE and HDPE were in the form of spherical granules with an average diameter of 3 mm. Recycled LPDE and HDPE were obtained from a plastic recycling company using municipal wastes (Doha Plastic Company). These were cylindrical granules with an average diameter of 3 mm and 4 mm for recycled LDPE and HDPE, respectively. The maximum size of the coarse aggregate (crushed limestone) and fine aggregates (river sand)^5^ were 9.5 mm and 2.3 mm, respectively. DRAMIX OL 6/16 straight cylindrical micro-steel fibre was used in all mixtures, with length and diameter, 6 mm and 0.16 mm respectively. The constituents of the different mixes are as follows (PC- Portland cement, FA-Fly ash, PM-Pozzolanic materials consisting of FA and Silica fume, SF-Steel fibre). The total binder is kept constant in each mix as 80% PC, 10% FA and 10% Silica fume (by weight of total binder). *Mix-1*(control sample): PM– 20%; *Mix-2*: PM-20%, SF-2%; *Mix-3*: PM-20%, SF-1%, recycled HDPE fibre–1%; *Mix-4*: PM-20%, SF-2%, virgin LDPE granule–10%; *Mix-5*: PM-20%, SF-2%, virgin HDPE granule–10%; *Mix-6*: PM-20%, SF-2%, recycled LDPE granule–10%; and *Mix-7*: PM-20%, SF-2%, recycled HDPE granule–10%. Further details on the sample preparations, physio-chemical chemical and microstructural properties of the concrete mixes can be found in a previous publication^5^.

The experiments were carried out on notched beam specimens prepared after the standard 28 and 90 days curing period^5^. All specimen had the dimensions of 160 × 40 × 40 mm (L × B × W), and notch length (*a*) of 0.4 *W* (Fig. 1a). The span length was 128 mm. The prefabricated notch had a width of 1 mm across the whole breadth (B) of the sample. Standard ASTM C78 three-point bend tests^22^ were conducted on the specimens (Fig. 1a) to determine the fracture toughness in conjunction with the evolution of birefringent measurements (recorded from the initial loading to the ultimate load level) using Photo-Stress Analysis Tomography (PSAT, Fig. 1b). Three specimens per mix were tested to monitor the level of statistical accuracy in the results. An epoxy-based birefringent coating of 300micron average thickness was applied uniformly on the polished surface of the beam including the notch area (bonded condition). The strain optic coefficient of the film is 0.06 m/m/(m/m), i.e., in the unit of retardation/thickness/strain^23^. The birefringent measurements were made using reflective PSAT^23, 24^ as illustrated in Fig. 1(b) (methods section). The shear stress concentration factor is also evaluated from the maximum shear stress distribution profiles displayed by the samples under the ultimate load (methods section). Standard ASTM C39 compression test^25^ were conducted for the different concrete samples to determine the Poisson’s ratio and elastic modulus, using cylindrical samples of dimension 200 mm length and 100 mm diameter. The flexural strength of the beams were evaluated using the ASTM C278 standard^22^.

## Results and Discussions

**Figure 2:**
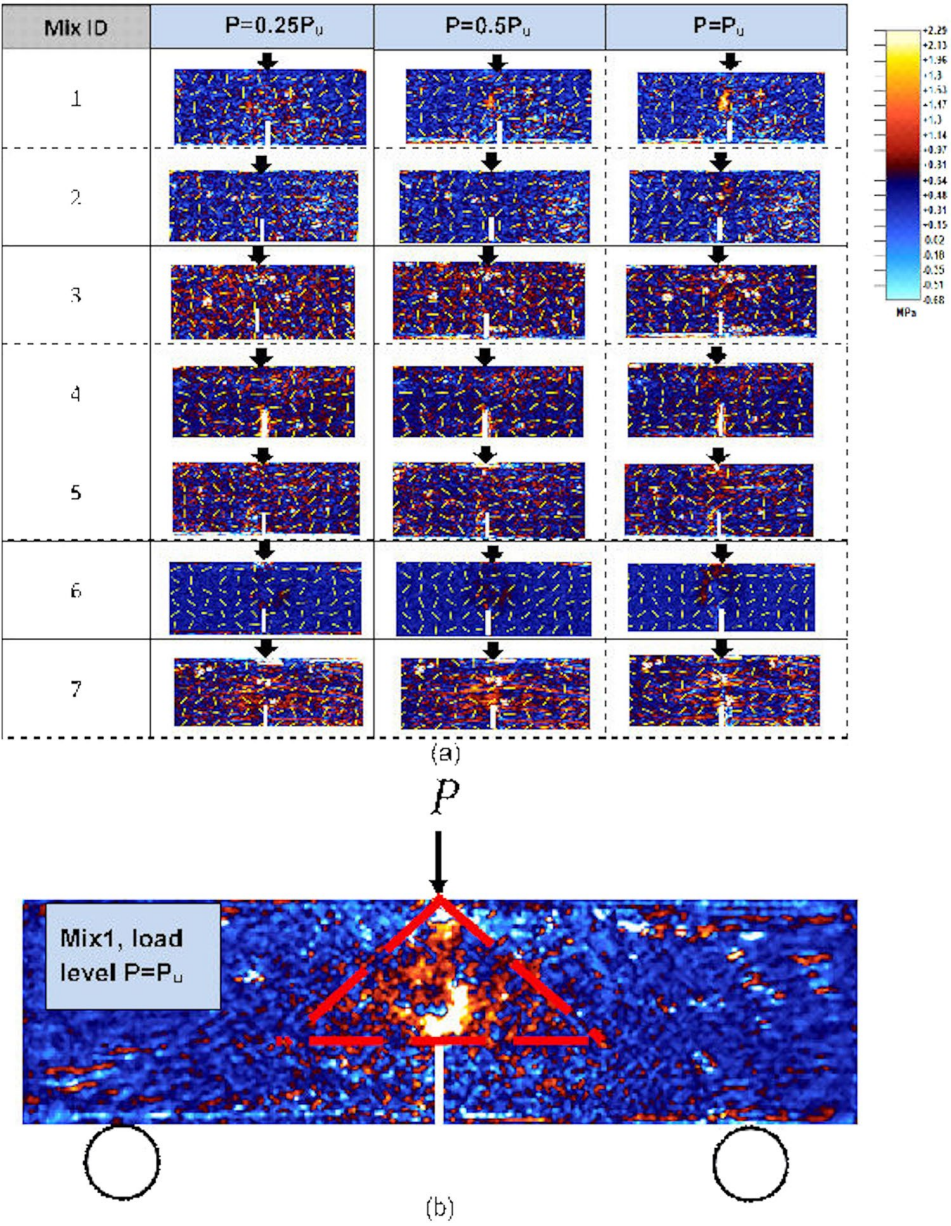
Maximum shear stress distribution on the concrete samples under different loading levels P. Pu is the ultimate load (strength) of the beam: (**a**) presented for the mid one-third region of the beams subjected to 28 days curing for comparison purposes. The arrows show the direction of the major principal stress and (**b**) a typical image for the whole beam subjected to 90 days curing. The triangular guidelines contain the region where maximum shear stress distribution is stronger (ahead of the notch) under the ultimate load. Plots similar to part (**a**) for all the mixes subjected to 90 days curing is provided in the supplementary document.

**Figure 3:**
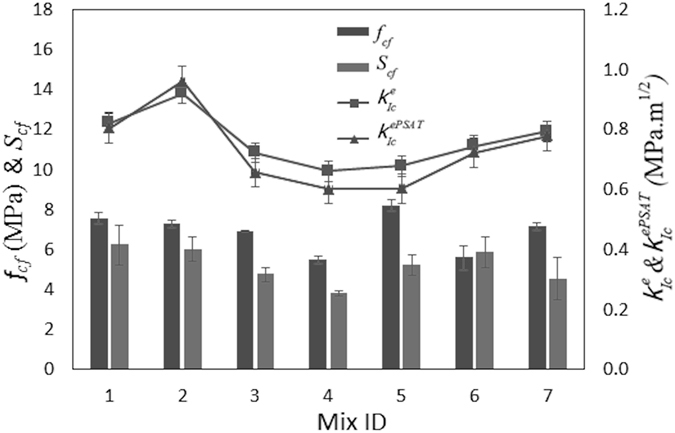
Flexural strength (${{\boldsymbol{f}}}_{{\boldsymbol{cf}}}$), shear stress concentration factor (***S***_***cf***_) and fracture toughness (${{\boldsymbol{K}}}_{{\boldsymbol{Ic}}}^{{\boldsymbol{e}}}$ and ${{\boldsymbol{K}}}_{{\boldsymbol{Ic}}}^{{\boldsymbol{ePSAT}}}$) for the concrete specimens subjected to 28 days curing.

The elastic modulus (effective modulus) for the different concrete mixes are presented in the Supplementary Table 1. The Poisson’s ratio of the samples was 0.256 ± 0.002, and also within the range for the plain and composite concretes reported in the previous studies^26^ with negligible differences between them. Figure 2(a) show the distribution of the maximum shear stress (*τ*_max_) in the 28 days cured beam samples ahead of the notch tip under the three-point bending tests. A similar plot for the 90 days cured samples are provided in the Supplementary Fig. 1. The plots show this profile for different load levels, however the images corresponding to the ultimate load are used in the post-processing software to locate the point of the maximum value of *τ*_max._ In other words, the location of maximum deviator stress under the ultimate load is identified to determine the effective crack length ahead of the notch tip (Supplementary Fig. 2), and subsequently the fracture toughness ***K***_***IC***_ and the energy release rate ***G***_***IC***_. For the different concrete samples (mixes), Fig. 3 shows the values of their fracture toughness, shear stress concentration factor (*S*_*cf*_) and flexural strength. In this, the current experiments-based fracture toughness value of the samples (${{\boldsymbol{K}}}_{{\boldsymbol{Ic}}}^{{\boldsymbol{ePSAT}}}$) are compared with corresponding predictions (${{\boldsymbol{K}}}_{{\boldsymbol{Ic}}}^{{\boldsymbol{e}}}$) based on the effective crack/notch model (methods section). However, further comparison of this based on the effective crack (notch) model taking into account the shear stresses in mode-1 (methods section) and the subsequent evaluations of the strain energy release rate for all the samples including the 90 days cured samples are summarised in the Supplementary Tables 2, 3. In general, they confirm a good level of agreements between current experimental results with the estimates based on the previous models.

It is worth remembering that the current experimental evaluation of fracture toughness did not use the shear stress concentration factor in the calculations, but uses the location of the maximum shear stress (highest deviator stress state) in measuring the effective crack length under the ultimate load. A closer examination of Fig. 3 suggests that useful correlations could be found between the shear stress concentration factor and the strength parameters (fracture toughness and flexural strength) of the concrete samples studied here. To examine this in detail and using the current experimental data of these quantities for 28 days curing (which is a standard practice in assessing concrete strength), a regression analysis is performed which resulted the following linear relation:

1 $${S}_{cf}={C}_{1}+{C}_{2}\,{f}_{cf}+{C}_{3}{K}_{Ic}^{ePSAT}$$

in which *C*_1_ = 7.88, *C*_2_ = 24.9 and *C*_3_ = 60.90.

**Figure 4:**
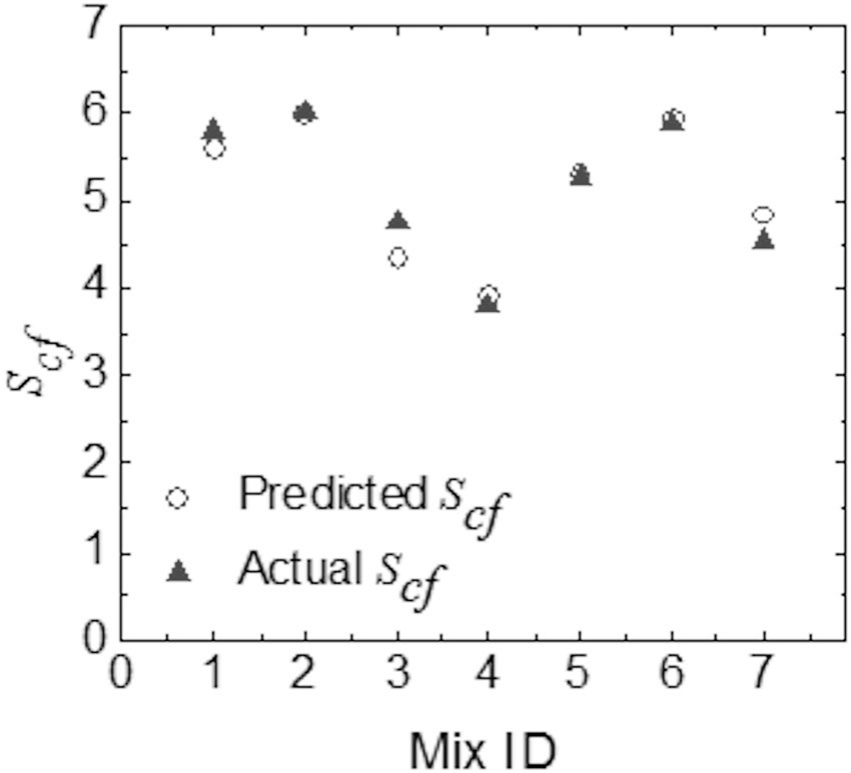
Actual (experimentally measured) shear stress concentration factor (*S*_*cf*_) is compared with that of the new model predictions.

From the regression analysis, R Square, Significance F and P values are obtained as 0.925, 0.006, and 0.01, respectively. As R square is closer to 1, and the significance F and P values are less than 0.05, this confirms the statistical reliance of Equation(1). Figure 4 shows the comparison between the experimentally measured shear stress concentration factor and its prediction using Equation(1), and they agree well considering the complexities of the different composite concrete mixes tested here.

### Conclusions and future scope

To conclude, we have shown an innovative method of assessing the fracture toughness of opaque heterogeneous concrete mixes in this work using the principles of photonics. A good level of correlation is found between the optically measured stress concentration factor of concrete, flexural strength and the fracture toughness of all the heterogeneous concrete mixes studied here. These could significantly help for standardising fracture strength testing of complex materials in future, when efforts to create sustainable engineering materials consisting of heterogeneous mixes, both natural and engineered, receives greater attention throughout the world. The current photonics research could be extended in future to account for other variables such as the loading rate effects and scale effects on the potential applications of photonic in assessing the fracture toughness and other strength characteristics of complex materials such as concretes with partially replaced recycled-wastes for different industrial applications. Furthermore, other experimental techniques such as digital image correlation (DIC)^27, 28^, and combined measurement techniques such as SEM(Scanning electron microscopy)-DIC^29^ and DIC-TSA(Thermal stress analysis)^30^ could be used to characterise the fracture processing zone of concrete mixes more effectively in future. This could help to get new information on the stress transmission and fracture characteristics of heterogeneous concrete mixes, which may not require to apply speckle patterns on the measurement side of the samples. Furthermore, with the recent advancements in the fabrication of speckle materials with the ability to sustain elevated temperatures (even well beyond 1000 °C)^31, 32^, DIC could be used to evaluate the characteristics of the fracture processing zones and fracture toughness of composite concretes as a function of different temperature environments in future.

## Methods

Standard ASTM C39 compression test^25^ for the different concrete samples were conducted to determine the Poisson’s ratio for which electrical strain gauges were mounted on the samples to measure the lateral and normal strains under the loading (supplementary Figure 3).

Flexural strength *f*_*cf*_ of the beams was calculated using the ASTM C293 standard^33^.

Photonic experiment: Initially the surface of the concrete specimens were polished well. An epoxy-based birefringent coating was applied uniformly (under a temperature environment of 21°) on the surface of the different concrete specimens^23, 34^. The coating was allowed to cure for 24 hours under the ambient temperature to obtain well-bonded coating on the samples (300microns average thickness). As the stiffness of the epoxy coating is much smaller than that of concrete, as well as the thickness of the coating was relatively small when compared with the characteristic dimensions of the concrete samples, the samples had no stiffness effect due to the presence of the coating on the concrete^1, 23^. We had also verified that the differences between the mechanical strength (e.g. *f*_*cf*_) of coated and uncoated concrete samples were negligible. Furthermore, the coatings were free from any shrinkage stresses in the photonic stress measurements as no birefringence was noticed in any of the unloaded specimens. The strain optic coefficient^23^ of the coating was 0.06 m/m/(m/m). Then the standard ASTM C78 three-point bend tests^22^ were conducted on the specimens (Fig. 1a) using a 500 kN Instron compression machine under a slow loading rate of 41 N/s (Supplementary Figure 4). We verified that, at no stages of the loading the coating delaminated. If the coating delaminates, it would be immediately obvious as the strain will not be passed into the coating and no photoelastic effect will be seen. Reflective grey field PSAT (Fig. 1b)^24^ was used in the current measurements. From the notch front, the birefringent response developed progressively with an increase in the loading level. The method of measuring birefringence on the surface of concrete is similar to measuring this on the surface of human cornea^24^, except that, as concrete is not naturally birefringent (unlike human eye), the birefringent coating applied on the surface of the concrete provided the similar functionality (/birefringence under loading). For practical purposes, the coating material (with a suitable strain optic coefficient^23^) can be chosen depending on the mechanical properties of the substrate material, and the strain measured on the coating pertains to that of the substrate. An expanded details on the working of PSAT can be found elsewhere^23, 35^. The wave length of light from the initial light source was 650 nm. In brief, a circularly polarised light sweeps on the surface of the loaded (birefringent-coated) specimen (Fig. 1b). Under the mechanical loading, the out coming retarded light from the sample is elliptically polarised^35^. This is characterised further using an analyser for different optical orientations of the analyser^35^. At the point of interest on the sample, the retardation can be related to the principal strain difference or to the principal stress difference using the well-known strain (or stress)-optic law^23, 36^. The difference between the principal strain components, and principal stress components are referred to as deviator strain and deviator stress respectively^19, 23^. The maximum shear stress (*τ*_max_) is equal to half the deviator stress^19, 23^. The shear stress concentration factor *S*_*cf*_ was evaluated under the ultimate load as^24^

2 $${S}_{cf}=\frac{{\tau }_{max}}{{\tau }_{avg}}$$

where *τ*_*avg*_ is the average value of the maximum shear stress calculated from the middle one-third area of the beam (a nominal stress measure and ignoring any effects due to the beam supports in the maximum shear stress calculations) as considered in Fig. 2a.

In the strength analysis of materials, fracture mechanics considers the evolution of the fracture process zone (FPZ) which is an energy-consuming zone occurring ahead of the crack (/notch) tip^13^. The characteristic depth of this plus the depth of the initial crack (/notch) size is considered as effective depth (Supplementary Fig. 2) in the treatment of evaluating the fracture toughness using the effective crack (notch) model^17^. The determination of the effective depth is not straightforward in the existing experimental testing of concretes. Locating the evolution of cracks, their geometrical features and the active energy processing regions ahead of the notch tip is difficult to measure with either a naked eye or using a conventional microscope to locate any visual crack processing zones in concrete testing. In the current experimental work, we sense the whole-filed distribution of the deviator stress (or maximum shear stress) ahead of the notch using the birefringent properties of the coated samples as explained above. At the grains scale, the slippage of the grains and subsequent formation of cracks under the mechanical loading is favourable to their deviator stress state (or related deviatoric state)^13, 19^. Hence in this study, we postulate that by sensing the evolution of *τ*_max_ (or the deviator stress), the location pertaining to the highest value of *τ*_max_ (or the point of the highest value of the retardation) from the initial notch tip under the ultimate load is used to evaluate the effective length of the crack (*a*_*e*_) and ${K}_{Ic}^{ePSAT}$. The fracture toughness is calculated as follows at first using the effective crack (notch) model^17^ (${K}_{Ic}^{e}$) and then ${K}_{Ic}^{ePSAT}$ by replacing the value of the theoretical effective crack length with the above said corresponding experimental data. The required equations are described briefly below^17^.

3 $${K}_{Ic}^{e}={\sigma }_{n}{a}_{e}^{1/2}Y(\frac{{a}_{e}}{W})$$

*σ*_*n*_ is the nominal tensile stress, given as follows:

4 $${\sigma }_{n}=\frac{6M}{B{W}^{2}}$$

*M* is the bending moment due to the self-weight of the beam and given by:

5 $$M=({P}_{max}+\frac{wS}{2})(\frac{S}{4})$$

$\frac{{a}_{e}}{W}$ is described theoretically as^17^;

6 $$\frac{{a}_{e}}{W}={C}_{1}{(\frac{{\sigma }_{n}}{E})}^{{C}_{2}}{(\frac{a}{W})}^{{C}_{3}}{(1+\frac{g}{W})}^{{C}_{4}}$$

where *w* is the self-weight of the beam per unit length, *C*_1_ = 0.249 ± 0.029, *C*_2_ = −0.120 ± 0.015, *C*_3_ = 0.643 ± 0.015 and *C*_4_ = 0.217 ± 0.073 and are coefficients obtained^17^ based on the regression analysis of the strength of concrete mix, *P*_*max*_ is the ultimate load, *E* is the effective elastic modulus of the concrete mix (specimen), *g* is the maximum aggregate size in the mix, *a* is the pre-existing crack length, *a*_*e*_ is the effective crack length. *W* is the depth of the beam (Fig. 1a).

7 $$Y(\alpha )=\frac{1.99-\alpha (1-\alpha )(2.15-3.93\alpha +2.7{\alpha }^{2})}{(1+2\alpha ){(1-\alpha )}^{3/2}}$$

In which $$\alpha =(\frac{{a}_{e}}{W})$$

The steps to calculate ${K}_{Ic}^{ePSAT}$ is same as that of ${K}_{Ic}^{e}$ explained above, except that $\frac{{a}_{e}}{W}$ ratio is substituted directly from the PSAT-based experimental measures as described earlier instead of using Equation (6). The fracture energy or strain energy release rate corresponding to ${K}_{Ic}^{e}$ and ${K}_{Ic}^{ePSAT}$ can be calculated respectively as follows^8, 37^:

8 $${G}_{I}^{e}=\frac{(1-{\nu }^{2}){K}_{Ic}^{e}}{E}$$

9 $${G}_{I}^{ePSAT}=\frac{(1-{\nu }^{2}){K}_{Ic}^{ePSAT}}{E}$$

where *v* is the Poisson’s ratio of the concrete mix (cylindrical specimen).

For comparison purposes, we have also used an additional model (results in the Supplementary Tables 2, 3): this is basically the effective crack (notch) model taking into account shear stresses in Mode 1 stress field (ECMS)^18^. This model considers that the stress field ahead of the pre-crack consists of tensile stress normal to the crack front, as well as an in-plane stress and shear stress^18^. The calculated fracture toughness and strain energy release rate using this model are denoted as ${\bar{K}}_{Ic}^{e}$ and ${\bar{G}}_{I}$ respectively. The related expressions are provided below^17, 18^;

10 $${\bar{K}}_{Ic}^{e}={\sigma }_{n}{a}_{e}^{1/2}{Y}_{1}(\alpha ){Y}_{2}(\alpha ,\beta )$$

11 $${Y}_{1}(\alpha )={A}_{0}+{A}_{1}\alpha +{A}_{2}{\alpha }^{2}+{A}_{3}{\alpha }^{3}+{A}_{4}{\alpha }^{4}$$

12 $${Y}_{2}(\alpha ,\beta )={B}_{0}+{B}_{1}\beta +{B}_{2}{\beta }^{2}+{B}_{3}{\beta }^{3}+{B}_{4}\alpha \beta +{B}_{5}{\beta }^{2}$$

The coefficients ${A}_{i}(i=0,1,\ldots ,4)$ and ${B}_{i}(\,j=0,1,\ldots ,5)$ are given below:

13 $$\begin{array}{rcl}{A}_{0} & = & 3.646,{A}_{1}=-6.789,\\ {A}_{2} & = & 39.240,{A}_{3}=-76.820,{A}_{4}=74.330\\ {B}_{0} & = & 0.4607,{B}_{1}=0.0484,{B}_{2}=-0.0063,\\ {B}_{3} & = & 0.0003,{B}_{4}=-0.0059,{B}_{5}=0.0033\,\\ {\bar{G}}_{I} & = & \frac{(1-{\nu }^{2}){\bar{K}}_{Ic}^{e}}{E}\end{array}$$

### Data Availability

All data generated or analysed during this study are included in this published article (and its Supplementary Information files).

## Electronic supplementary material

Manuscript supplementary document
