# Supplementary material for: Photonics and fracture toughness of heterogeneous composite materials
Source: Sci Rep. 2017 Jul 3;7:4539. doi: 10.1038/s41598-017-04782-7 (PMC5495800; doi:10.1038/s41598-017-04782-7)
Supplement: Supplementary file 1 — Manuscript supplementary document [file 41598_2017_4782_MOESM1_ESM.pdf]

Manuscript title: Photonics and fracture toughness of heterogeneous composite materials

S. Joseph Antony[1*], George Okeke[1], D. Deniz Tokgoz [2] and N. Gozde Ozerkan[2]

[a] School of Chemical and Process Engineering, University of Leeds, Leeds LS2 9JT, UK

[b] Center for Advanced Materials, Qatar University, P.O. Box 2713, Doha, Qatar

Supplementary document:

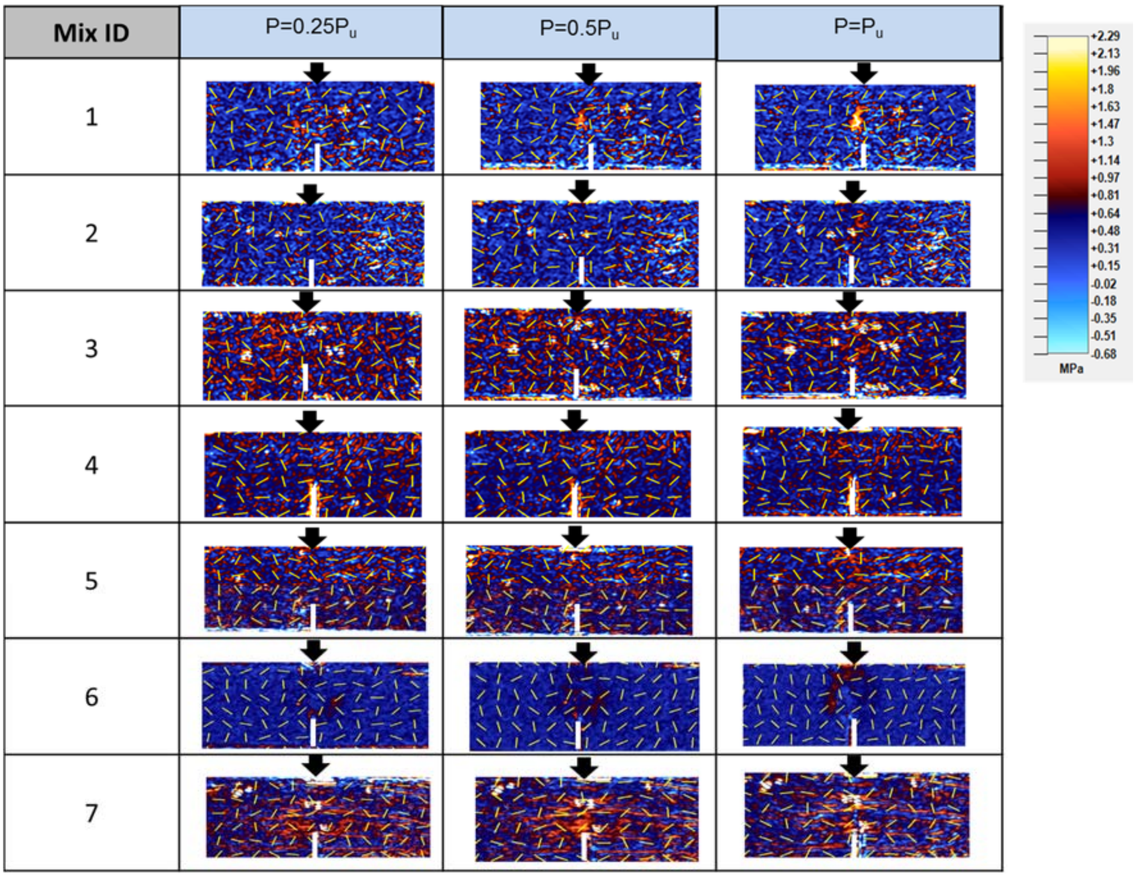

Supplementary Figure 1 Maximum shear stress distribution on the concrete samples under different loading levels P subjected to 90 days curing for the mid one-third region of the beams. Pu is the ultimate load (strength) of the beam.

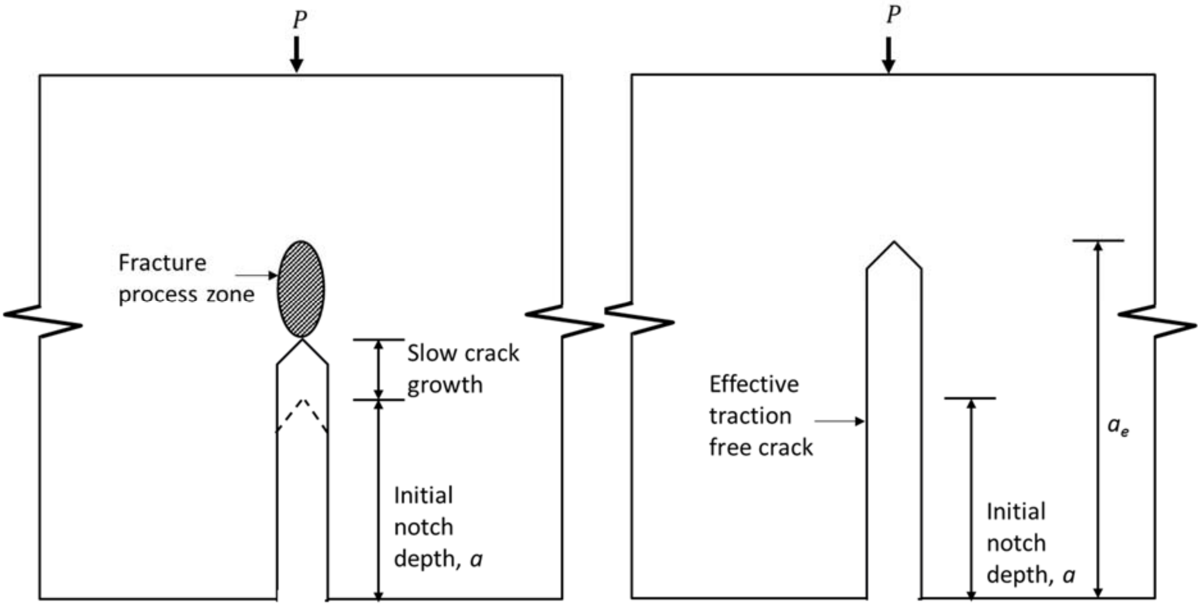

Supplementary Figure 2 The concepts of effective notch depth $\boldsymbol{a_e}$, and the equivalent measure using PSAT is $\boldsymbol{a_{ePSAT}}$ [17]

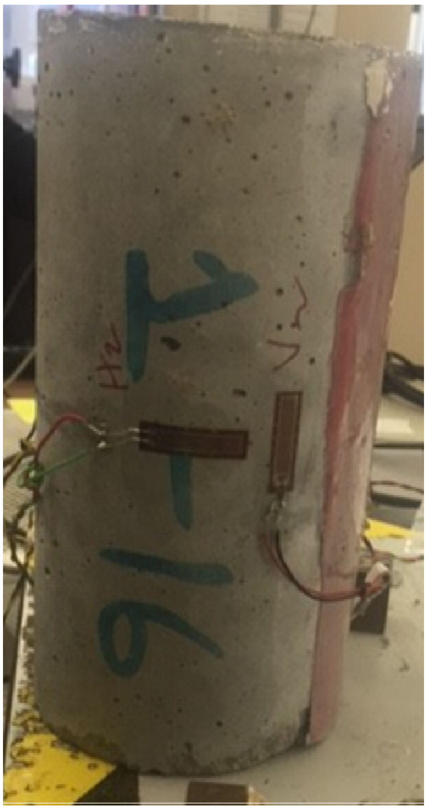

Supplementary Figure 3 A typical concrete sample on which electrical strain gauges are mounted to measure the lateral and normal strains under the loading

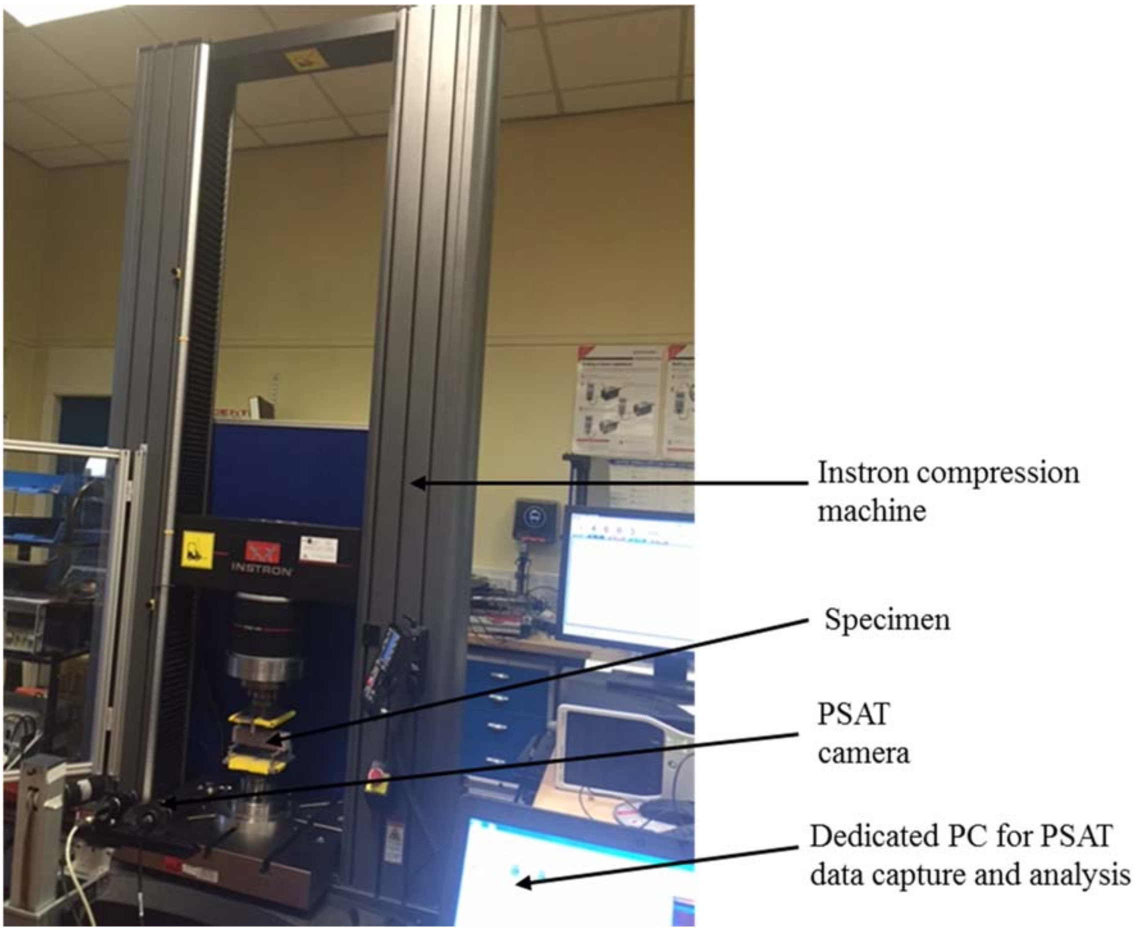

Supplementary Figure 4 A notched concrete specimen subjected to the Standard ASTM C78 three-point bend tests. PSAT camera is used to track the evolution of the maximum shear stress distribution on the surface of the specimen.

Table 1 Elastic modulus E (GPa) for different mixes of 28 and 90 days cylinders

Mix ID	28 days	90 days
1	25.47±0.44	83.35±0.79
2	28.48±0.77	30.07±0.50
3	26.53±0.26	33.00±0.48
4	18.96±0.79	23.62±0.41
5	19.68±0.31	32.28±0.39
6	21.27±0.32	27.30±0.68
7	29.61±0.31	32.05±0.69

Table 2 Flexural strength, fracture toughness, and fracture energy for 28 days specimens

Mix ID	Flex. strength (MPa)	$K_{Ic}^{e}(MPa\, m^{1/2})$	$\bar{K}_{Ic}^{e}(MPa\, m^{1/2})$	$K_{Ic}^{ePSAT}(MPa\, m^{1/2})$	$G_{I}(J\, m^{-1})$	$\bar{G}_{I}(J\, m^{-1})$	$G_{I}^{ePSAT}(J\, m^{-1})$
1	7.55±0.29	0.82±0.01	1.05±0.01	0.80±0.01	26.51±0.36	24.77±0.34	26.51±0.36
2	7.27±0.21	0.92±0.03	1.12±0.04	0.96±0.03	32.51±0.36	30.37±0.34	34.14±0.81
3	6.92±0.03	0.72±0.02	0.94±0.03	0.66±0.05	19.73±0.21	18.44±0.20	16.41±0.19
4	5.48±0.19	0.66±0.02	0.84±0.03	0.60±0.05	23.17±0.65	21.65±0.60	19.44±1.16
5	8.21±0.30	0.68±0.03	0.87±0.03	0.60±0.04	23.49±0.62	21.95±0.58	18.69±1.10
6	5.59±0.62	0.75±0.03	0.95±0.04	0.72±0.03	26.27±0.56	24.54±0.53	26.32±0.74
7	7.15±0.19	0.79±0.04	1.04±0.05	0.78±0.05	21.41±0.14	20.00±0.13	20.64±0.61

Table 3 Flexural strength, fracture toughness, and fracture energy for 90 days specimens

Mix ID	Flex. strength (MPa)	$K_{Ic}^{e}(MPa\, m^{1/2})$	$\bar{K}_{Ic}^{e}(MPa\, m^{1/2})$	$K_{Ic}^{ePSAT}(MPa\, m^{1/2})$	$G_{I}(J\, m^{-1})$	$\bar{G}_{I}(J\, m^{-1})$	$G_{I}^{ePSAT}(J\, m^{-1})$
1	7.99±0.20	0.84±0.07	1.23±0.09	0.84±0.00	8.52±0.02	7.96±0.02	8.44±0.03
2	7.31±0.07	0.98±0.01	1.26±0.01	0.99±0.03	32.00±0.83	29.90±0.77	32.75±1.80
3	7.50±0.30	0.59±0.07	0.81±0.08	0.53±0.15	10.98 ±0.12	10.26±0.11	14.19±3.98
4	5.64±0.35	0.51±0.02	0.68±0.02	0.38±0.02	10.92±0.30	10.20±0.28	6.11±0.19
5	5.59±0.24	0.76±0.07	1.01±0.08	0.73±0.10	18.31±1.45	17.11±1.35	21.24±1.83
6	5.66±0.09	0.56±0.04	0.75±0.05	0.48±0.06	11.69±1.14	10.93±1.06	12.37±1.40
7	6.98±0.38	0.84±0.04	1.10±0.05	0.75±0.02	22.22±0.68	20.76±0.64	17.62±0.63
